# Kaplan anastomosis of the ulnar nerve: a case report

**DOI:** 10.1186/1752-1947-2-107

**Published:** 2008-04-15

**Authors:** Georgios Paraskevas, Christos Ch Gekas, Alexandros Tzaveas, Ioannis Spyridakis, Alexandra Stoltidou, Parmenion Ph Tsitsopoulos

**Affiliations:** 1 Department of Anatomy, Medical Faculty, Aristotle University of Thessaloniki, Thessaloniki, Greece

## Abstract

**Introduction:**

The sensory innervation of the hand is usually unvarying and anomalies in this area are uncommon.

**Case presentation:**

We report the case of a rare ulnar nerve branch called a Kaplan anastomosis, which anastomosed the dorsal cutaneous branch with the ulnar nerve prior to its bifurcation into the superficial and deep ramus.

**Conclusion:**

Many authors have reported unusual ulnar nerve branches and knowledge of these anatomical variations is important for the interpretation of pain and sensory loss in the area sustained during injuries or surgical procedures. Our finding is the fourth case of a Kaplan anastomosis to be described in the literature.

## Introduction

Knowing that there is a nerve variation in the ulnar area of the hand is important and could explain sensory loss or pain in patients following surgical procedures or trauma. Ulnar nerve variations are consistently located in the origin or course of the distal branches. The communicating branches between the ulnar and median nerve have been described mostly in the hand and arm. A communicating branch of the dorsal and superficial ramus of the ulnar nerve, known as a Kaplan anastomosis, is rare and this is the fourth case to be described in the literature.

## Case presentation

**Figure 1:**
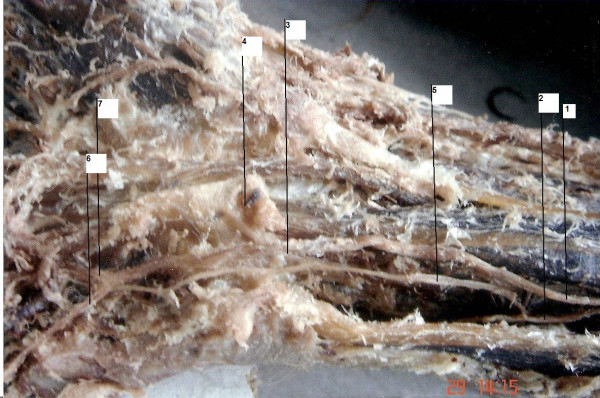
**View of the cadaveric finding of a Kaplan anastomosis.** (1) Ulnar nerve, (2) dorsal cutaneous branch of the ulnar nerve, (3) Guyon's canal, (4) flexor retinaculum of the wrist, (5) Kaplan anastomosis, (6) superficial ramus, (7) deep ramus of the ulnar nerve.

**Figure 2:**
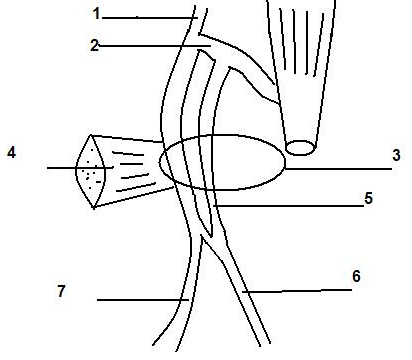
Schematic presentation.

We dissected an upper limb of a 76-year-old male cadaver, for educational purposes. First, we exposed and then removed the fascia of the forearm and palm. The ulnar nerve and its dorsal ramus were exposed after reflecting the flexor carpi ulnaris tendon medially, by removing the flexor retinaculum and transecting the roof of Guyon's canal. The ulnar artery was removed in order to obtain a better view of the course of the ulnar nerve in this region. After careful dissection in the wrist area, we found a thin nerve branch, originating from the dorsal ulnar nerve branch approximately 3 cm proximal to the ulnar styloid process. This nerve branch, called Kaplan anastomosis, was parallel to the ulnar nerve. This nerve branch gave off many thin branches along its course for the synovial membrane of the joint, the abductor of the fifth finger and the skin of the area. The nerve branch then passed through Guyon's canal close to the pisiform bone and finally merged with the trunk of the ulnar nerve just before its division into the superficial and deep rami (Figures 1 and 2).

The normal course of the ulnar nerve in the forearm is between the flexor carpi ulnaris and the flexor digitorum profundus. The dorsal branch, which is responsible for the sensory innervation of the medial half of the hand and half of the digits, arises approximately 5 cm proximally to the wrist. In the hand, the ulnar nerve enters Guyon's canal underneath the superficial part of the flexor retinaculum and is divided into a superficial (sensory) and deep (motor) branch [1].

Anomalies of the sensory innervation of the hand are uncommon [2]. In 1963, Kaplan described a nerve branch that arose from the dorsal cutaneous branch of the ulnar nerve and finally merged with the superficial ramus of the ulnar nerve [3]. Similarly, Wulle [4] described a case of Kaplan anastomosis that merged with the superficial ramus of the ulnar nerve.

Hoogbergen and Kauer [5] also found a Kaplan anastomosis. In particular, they found a significant case of a Kaplan anastomosis emerging from the dorsal cutaneous branch of the ulnar nerve approximately 2.5 cm proximally to the ulnar styloid process. It gave off three branches along its course. The first branch emerged just proximally to the ulnar styloid process and ran towards the radiocarpal joint. It was located between the abductor digiti minimi and the deep ramus of the ulnar nerve where the Kaplan anastomosis gave off two branches. One branch ran towards the abductor digiti minimi muscle, while the other ran towards the fifth carpometacarpal joint. It is of interest that eventually the Kaplan anastomosis merged with the deep ramus of the ulnar nerve [5].

Our case differs from previous reported cases of Kaplan anastomoses in that the nerve is united with the trunk of the ulnar nerve just before its bifurcation into the superficial and deep ramus. Naturally we must distinguish between Kaplan anastomoses and dorsal cutaneous branch variations or ulnar-median nerve communicating branches. Paul et al [6] reported a rare finding of the dorsal branch of the ulnar nerve which began in the upper quarter of the forearm and divided into medial and lateral branches. The medial branch merged from the hypothenar region with the deep branch from the ulnar nerve and the lateral branch became cutaneous. The communication branches between the ulnar and the median nerve are well recognized and the majority of cases are directed from the ulnar to the median nerve [7,8]. However, in most cases, these communication branches affect the sensory innervation of the digits.

Many authors have reported sensory communication branches between the ulnar and median nerves in the hand [7]. In 1991, Ferrari and Gilbert classified them [9]. Most authors found the incidence of communication branches between the ulnar and the median nerve to be over 90%, so this kind of anatomical variation should be considered as normal [10].

## Conclusion

Knowing of the existence of the Kaplan anastomosis of the ulnar nerve, which is a rare anatomical variation, is important because this branch can be damaged in cases of pisiform fracture, in surgery of the pisiform bone and flexor carpi ulnaris, in trauma in this area and in conditions which involve Guyon's canal. As Kaplan states, the presence of these anastomoses must be noted when pain or sensory disorders in the pisiform area appear after trauma or surgery [3]. As many authors have reported the existence of communication branches between the ulnar and median nerve, the existence of Kaplan anastomoses and other unusual branches of the ulnar nerve must be considered normal.

## Competing interests

The author(s) declare that they have no competing interests.

## Authors' contributions

GP, CCG and AT found the Kaplan anastomosis during the dissection of the hand. IS and AS were involved in reviewing the literature. PPT was involved in the research on the importance of the finding and its interpretation. GP was also the main author and responsible for final proofreading of the article. All authors read and approved the final manuscript.

## Consent

Written informed consent was obtained from the patient's next-of-kin for publication of this case report and accompanying images. A copy of the written consent is available for review by the Editor-in-Chief of this journal.
